# Supplementary material for: Evidence supported by Mendelian randomization: impact on inflammatory factors in knee osteoarthritis
Source: Front Med (Lausanne). 2024 May 28;11:1382836. doi: 10.3389/fmed.2024.1382836 (PMC11165061; doi:10.3389/fmed.2024.1382836)
Supplement: Supplementary file 1 [file Data_Sheet_1.ZIP › data/GCST90274790_5e6/again/790.leaveoneout.pdf]

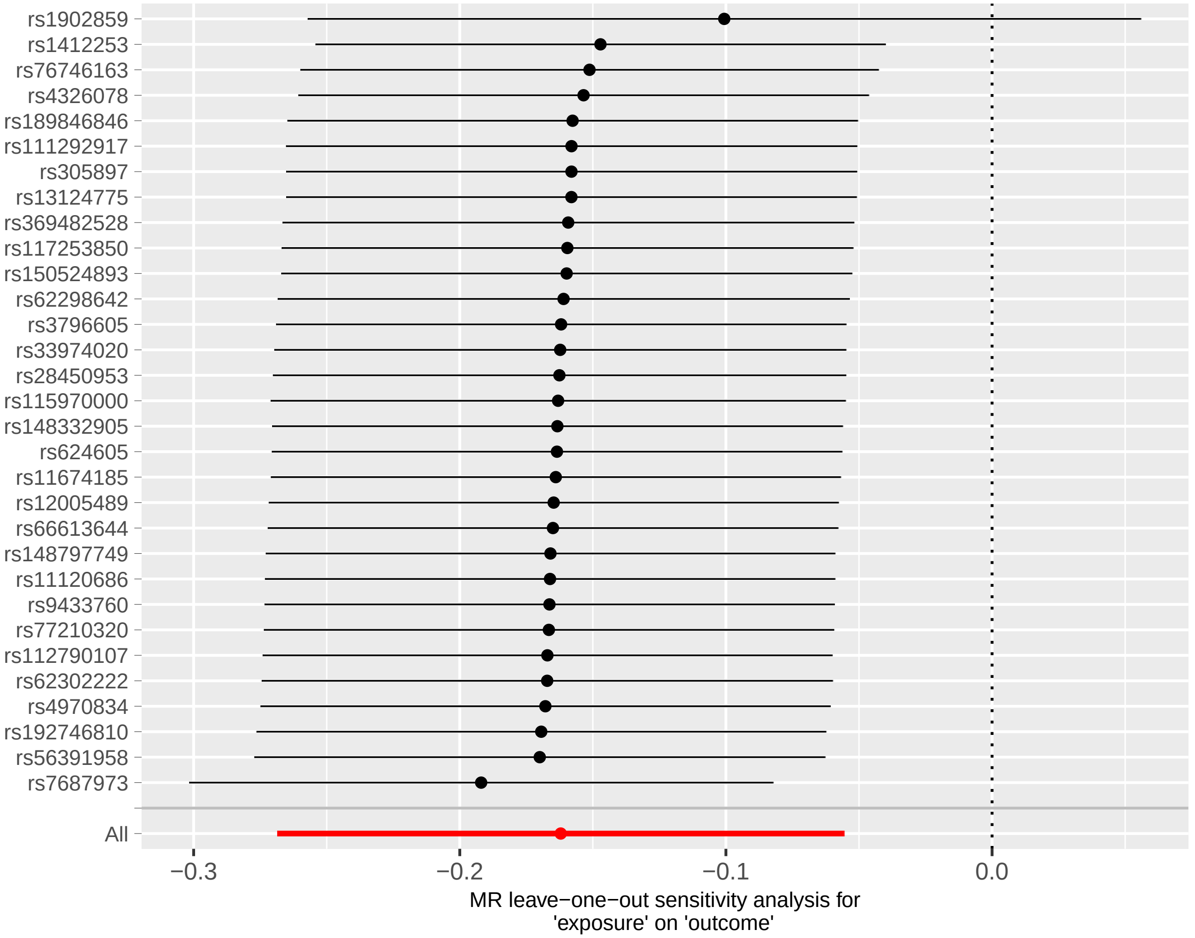
rs1902859
rs1412253
rs76746163
rs4326078
rs189846846
rs111292917
rs305897
rs13124775
rs369482528
rs117253850
rs150524893
rs62298642
rs3796605
rs33974020
rs28450953
rs115970000
rs148332905
rs624605
rs11674185
rs12005489
rs66613644
rs148797749
rs11120686
rs9433760
rs77210320
rs112790107
rs62302222
rs4970834
rs192746810
rs56391958
rs7687973
All
−0.3
−0.2
−0.1
0.0
MR leave−one−out sensitivity analysis for
'exposure' on 'outcome'
